# Supplementary material for: Tidewater-glacier response to supraglacial lake drainage
Source: Nat Commun. 2022 Oct 14;13:6065. doi: 10.1038/s41467-022-33763-2 (PMC9568665; doi:10.1038/s41467-022-33763-2)
Supplement: Supplementary file 1 — Supplementary Information [file 41467_2022_33763_MOESM1_ESM.pdf]

Supplementary Information for

Tidewater-glacier response to supraglacial lake drainage

Laura A. Stevens[1†], Meredith Nettles[2], James L. Davis[2], Timothy T. Creyts[2], Jonathan Kingslake[2], Ian J. Hewitt[3], and Aaron Stubblefield[4]

[1]Department of Earth Sciences, University of Oxford, Oxford, UK
[2]Lamont-Doherty Earth Observatory of Columbia University, Palisades, NY, USA
[3]Mathematical Institute, University of Oxford, Oxford, UK
[4]Thayer School of Engineering, Dartmouth College, Hanover, NH, USA

[†]Corresponding Author: Laura A. Stevens (laura.stevens@earth.ox.ac.uk)

Contents:

1. Supplementary Methods
2. Supplementary Figures 1–19
3. Supplementary Table 1

1. Supplementary Methods

Stochastic-filter analysis of glacier surface positions. GPS data were processed in kinematic mode using the TRACK software package[1] to yield 15-s position estimates[2–5]. Here, we eliminate position estimates with unfixed biases, and rotate the time series to obtain position estimates in the local along-flow direction at each station. We use a stochastic-filter approach[6,7] to obtain time-dependent velocity estimates in the local along-flow direction at each GPS site, following the methodology of Davis et al.[4] and Stevens et al.[5]. Helheim Glacier flow is known to respond to both diurnal melt forcing and tidal forcing, and to change speed abruptly at times associated with glacial earthquakes[2–5]. We model along-flow glacier position as the sum of three separate processes: (1) the time-integrated mean flow speed, $\int_{t_\circ}^{t} v(t')dt'$ over the epoch $t_\circ$ to t; (2) a response to forcing by the ocean tides, $A(t)F\big(t-\tau(t)\big)$; and (3) a diurnal variation in position, $x_D(t)$. A contribution from noise in the position estimates, $\epsilon(t)$, is also allowed, such that the full model can be written

$$x(t) = x_\circ + \int_{t_\circ}^{t} v(t')dt' + A(t)F\big(t-\tau(t)\big) + x_D(t) + \epsilon(t) \qquad , \qquad (1)$$

where $x_\circ = x(t_\circ)$ is the position at the initial epoch $t_\circ$, $A(t)$ is the amplitude of the tidal admittance, $F(t)$ is the tide height, and $\tau(t)$ represents a lag in the glacier response to the tide. Following previous work, step changes in along-flow velocity $v(t)$ are allowed at the times of glacial earthquakes, which represent large calving events[2,4]. Our implementation of the stochastic filter is identical to that of Stevens et al.[5], with the exception that we use a slightly larger random-walk variance rate (0.1 m^2 d^{-1}) for the along-flow velocity term $v(t)$. This adjustment results in a slightly more temporally variable estimate of v than in ref. 5, which allows for more precise identification of the three time points t_0, t_{peak}, and t_{node} that we use to characterize the velocity response to lake drainage.

Based on previous results using the same GPS data[3,4], we describe the response of the glacier position to the ocean tide using the linear admittance representation $A(t)F\big(t-\tau(t)\big)$, where the amplitude of the tidal admittance $A(t)$ is a scalar relating the amplitude of the ocean tide and the glacier response, and $\tau(t)$ represents a lag in the glacier response. The tide height $F(t)$ is given by the AOTIM-5 model[8], which was validated for this region using local observations[3,4]. Previous results show no significant tidal response for stations more than ~10 km from the calving front (i.e., IS26–29), and we therefore fix $A(t)$ to zero at those stations. The diurnal modulation of glacier position, $x_D(t)$, is represented as $x_D(t) = a_c(t)\cos 2\pi f_o t + a_s(t)\sin 2\pi f_o t$, where f_o is one cycle per day. The amplitudes $a_s(t)$ and $a_c(t)$ are modeled

as slowly varying random-walk stochastic processes with a variance rate of 2×10^{-5} m^2 d^{-1} (ref. 5).

Results of this velocity-estimation approach are shown for all stations recording usable data during the lake-drainage event in Supplementary Figures 9–17. In Supplementary Figure 9 for station IS35, panel (a) shows the along-flow component of station position from 2007/222–235; the station moves more than 300 m during this time. Panel (b) shows the detrended position, where the trend removed represents the average glacier speed at this site during the observing period (the trend seen in panel (a)). Panel (c) shows $v(t)$ estimated from Equation 1, where the speed is given with respect to the mean speed of 23.2 m d^{-1}. Step changes in speed associated with glacial earthquakes occur near the end of day 225. Because this station is located near the glacier terminus, the data show a tidal modulation of flow. The time-varying tidal admittance and lag parameters, $A(t)$ and $\tau(t)$, are shown in panels (d) and (e), respectively. The estimated tidally modulated component of flow, $A(t)F\big(t-\tau(t)\big)$, is shown in panel (f). This station also shows diurnally modulated flow. The time-varying diurnal parameters, $a_c(t)$ and $a_s(t)$, are shown in panels (g) and (h), respectively, and the full diurnal position signal, $x_D(t)$, is shown in panel (i). Finally, the time-varying residual, $\epsilon(t)$, is shown in panel (j). Gaps in the data visible in panels (a) and (j) arise from the elimination of noisy data (i.e., those data with biases unfixed in the TRACK position estimates). Short-duration, low-amplitude excursions in the $v(t)$, $A(t)$, $a_c(t)$, and $a_s(t)$ parameters (e.g., Supplementary Fig. 14) are likely the result of multipathing and/or ionospheric disturbances[9]. The modeling approach successfully isolates the periodic variability in the glacier flow, such that neither the velocity term $v(t)$ nor the residual $\epsilon(t)$ show substantial remaining periodicity.

The stations located on the lower glacier terminus—IS35, IS36, IS38, IS39, and IS25—include estimates of the tidal-modulation signal (Supplementary Figs. 9–13). At stations IS26–IS29, where no tidal-modulation signal is present in the GPS position estimates[5], the term $A(t)$ is fixed to zero (Supplementary Figs. 14–17). Along-flow velocity $v(t)$ at station IS25 (Supplementary Fig. 13c) during the time of the lake drainage (2007/229–232) is consistent with $v(t)$ at the other stations, but, we do not attempt to identify lake-drainage time points at station IS25 owing to a deterioration in data quality beginning on 2007/227 (Supplementary Fig. 13a,j). Two additional stations in operation during the drainage event (IS30, IS31), located inland of station IS29, recorded data of insufficient quality (*i.e.,* biases were not fixed for more than half of the position estimates on each day) for high-resolution, stochastic-filter analysis during this time period.

2. Supplementary Figures

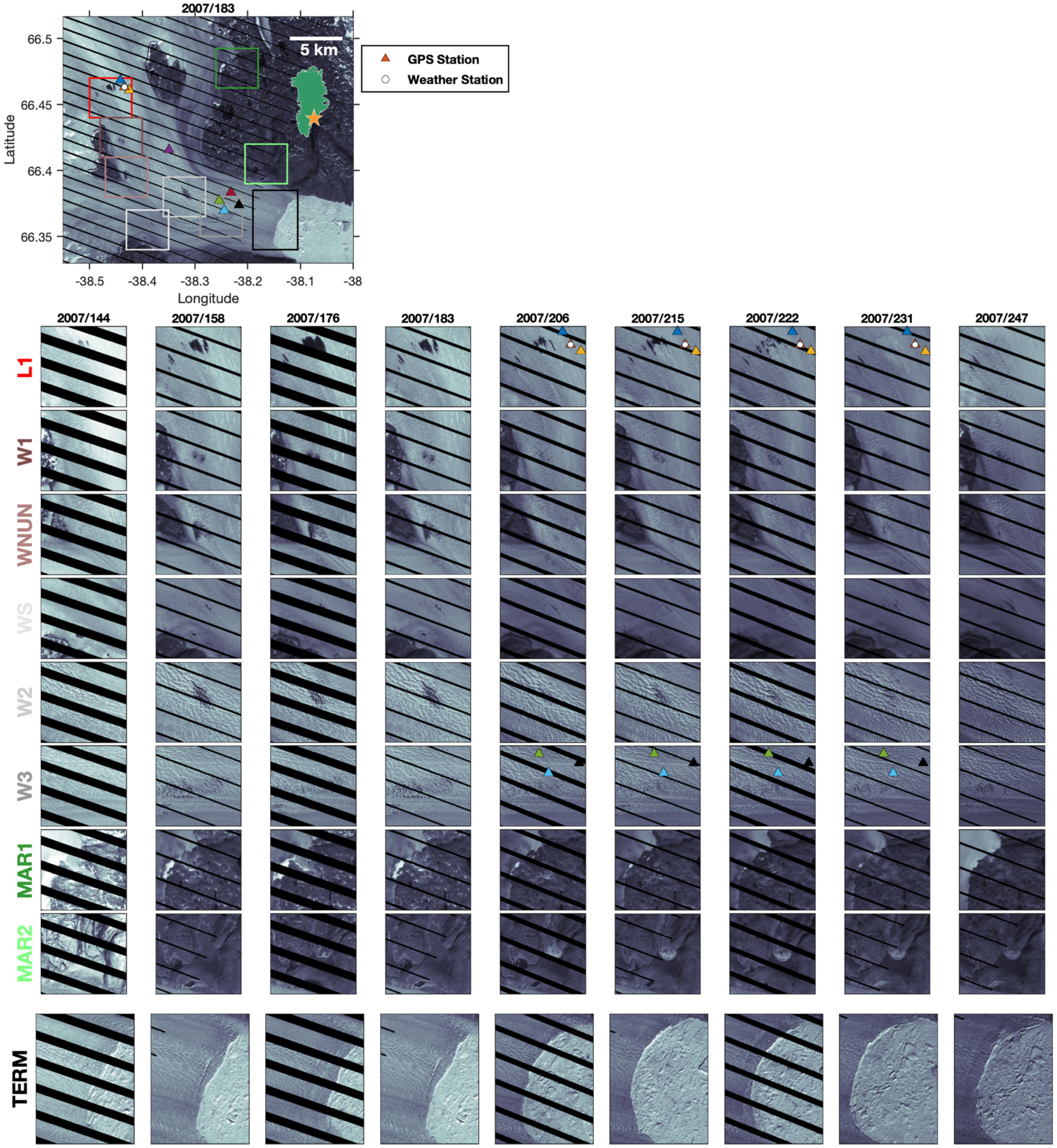

Supplementary Figure 1: Landsat images of Helheim Glacier supraglacial lakes and terminus during the 2007 melt season. Images of all identified supraglacial lakes and two marginal lakes along Helheim Glacier in Landsat images between 2007/144 and 2007/247. Images are available approximately bi-weekly. Boxes in top map identify six supraglacial water bodies ((red) L1, (dark brown) W1, (light brown) WNUN, (light grey) WS, (medium grey) W2, and (dark grey) W3) and two regions with marginal lakes ((dark green) MAR1 and (bright green) MAR2). The terminus region is also shown: (black) TERM. Where applicable, water body names are equivalent to the names used in Everett et al.[10]. GPS and AWS stations are shown on images when the GPS and/or AWS were in operation. Black diagonal lines on images are due to satellite malfunction; the black lines are of varying widths between sequential images and across regions in the same image. Landsat images courtesy of the U.S. Geological Survey.

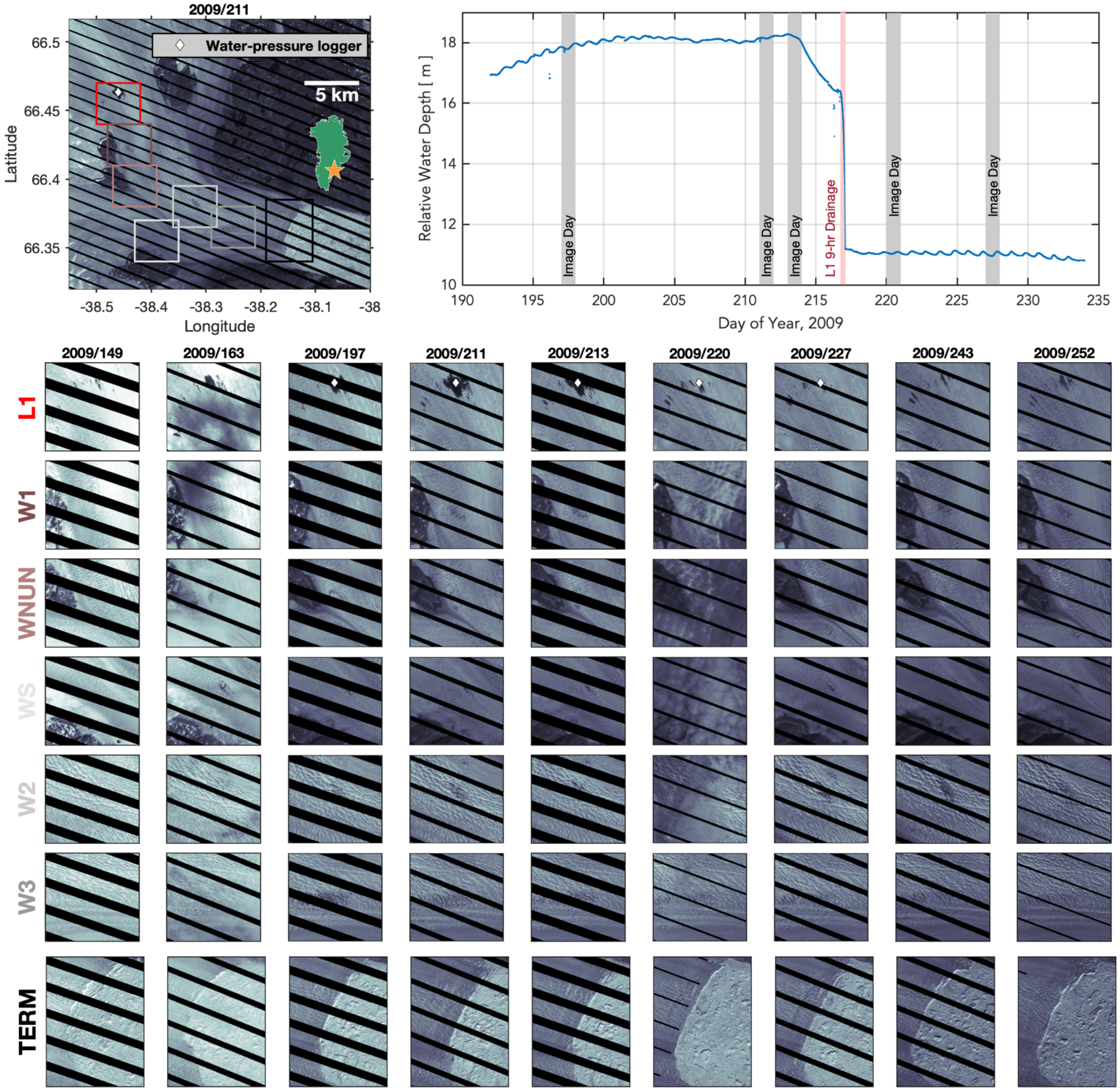

Supplementary Figure 2: Landsat imagery of Helheim Glacier supraglacial lakes and terminus during the 2009 melt season. Panels are as in Supplementary Figure 1, but for the 2009 melt season. Clouds obscure portions of the 2009/163 and 2009/220 images. Timeseries at upper right shows (blue) water depth above a pressure transducer deployed in L1 at the location shown by the white diamond in L1 images. Pressure-transducer data show an L1 drainage duration of 9 hours (pink vertical bar). Days with Landsat images are shown with grey vertical bars. Source data are provided as a source data file. Landsat images courtesy of the U.S. Geological Survey.

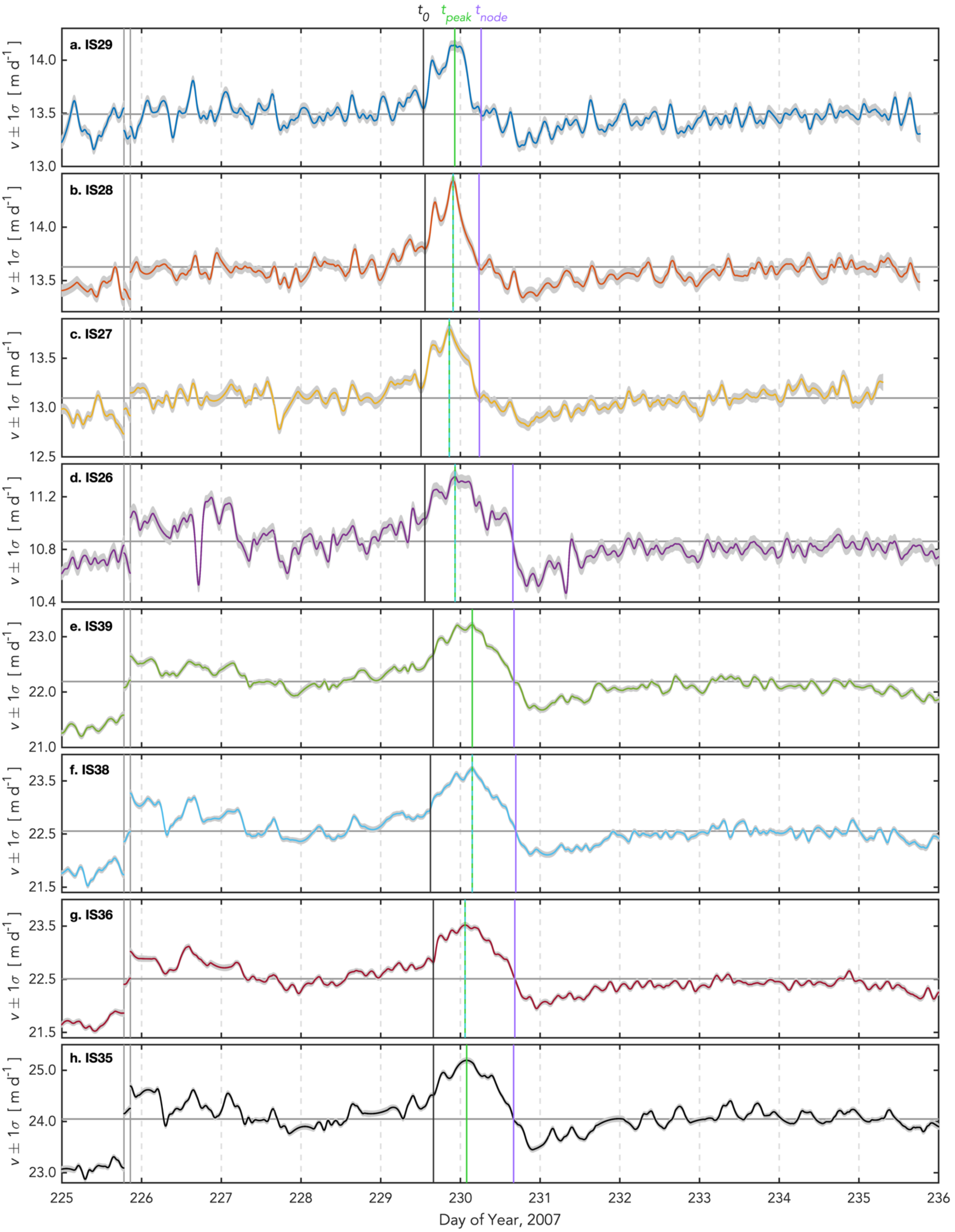

Supplementary Figure 3: Along-flow velocity v during supraglacial lake drainage. Grey shading shows ±1σ error bounds. Solid grey vertical lines on 2007/225 represent times of glacial earthquakes (large calving events), when v is allowed to be discontinuous in the stochastic filter[4,5]. The time of (black) t_0; (green) t_{peak}; and (purple) t_{node} observed at each GPS station are shown with vertical lines. Average v on 2007/228, v_{228}, is shown with a horizontal grey line. Source data are provided as a source data file.

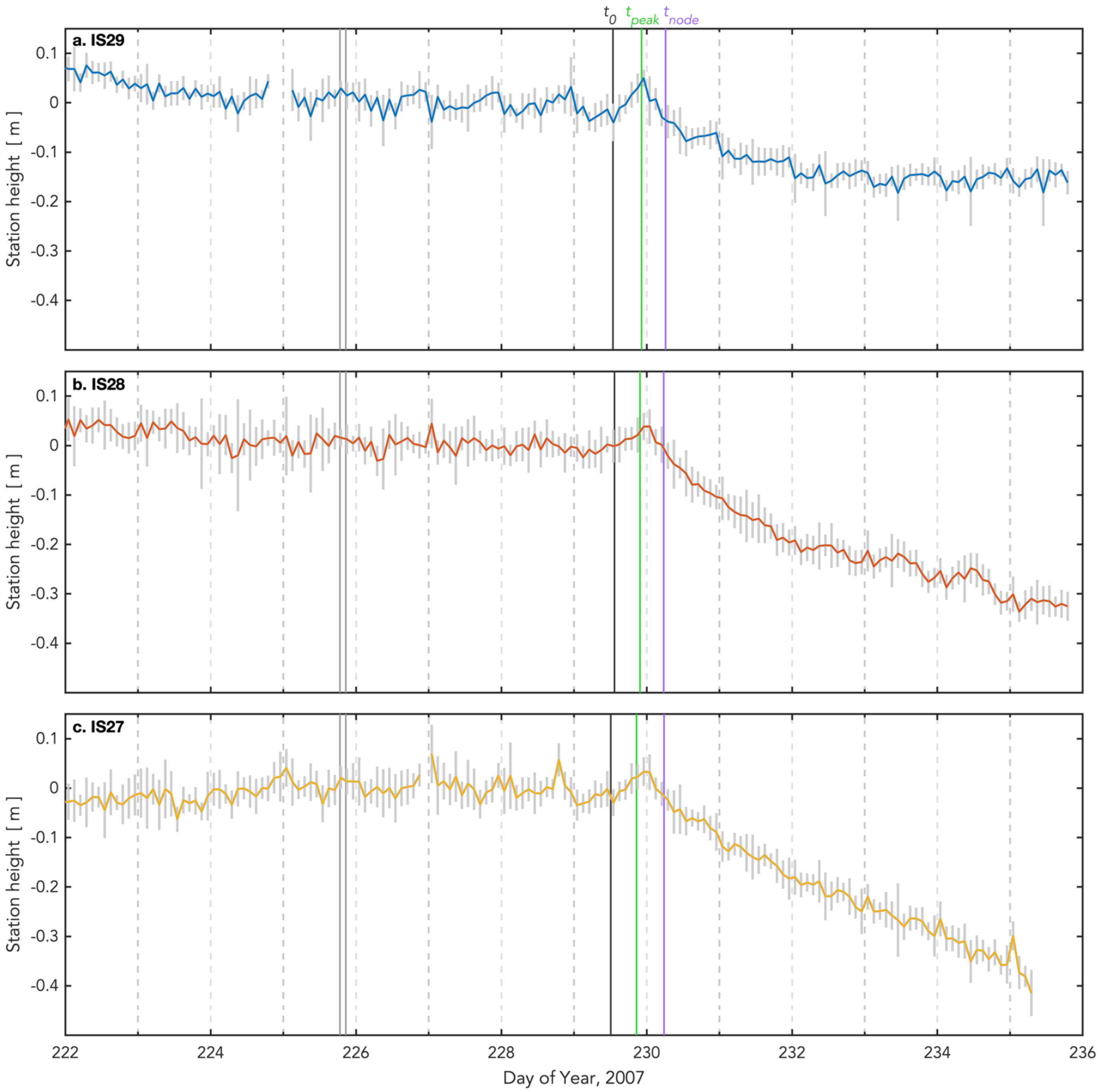

Supplementary Figure 4: GPS station height during supraglacial lake drainage. Timeseries show mean height over a 2-hr time window after removal of the mean and trend for the period 2007/226.0–229.0. Vertical grey bars show ±2 standard deviations of the distribution of height estimates during each 2-hr window. Solid grey vertical lines on 2007/225 represent times of glacial earthquakes (large calving events). The time of (black) t_0, (green) t_{peak}, and (purple) t_{node} for each station (Methods; Supplementary Fig. 3) are shown with vertical lines. The lake-proximal stations IS27–29 (**a–c**) show ~0.06 m of uplift during the lake drainage. The lower-elevation stations (not shown), located within 5 km of the terminus, show no anomalous vertical surface displacement during the lake drainage. Source data are provided as a source data file.

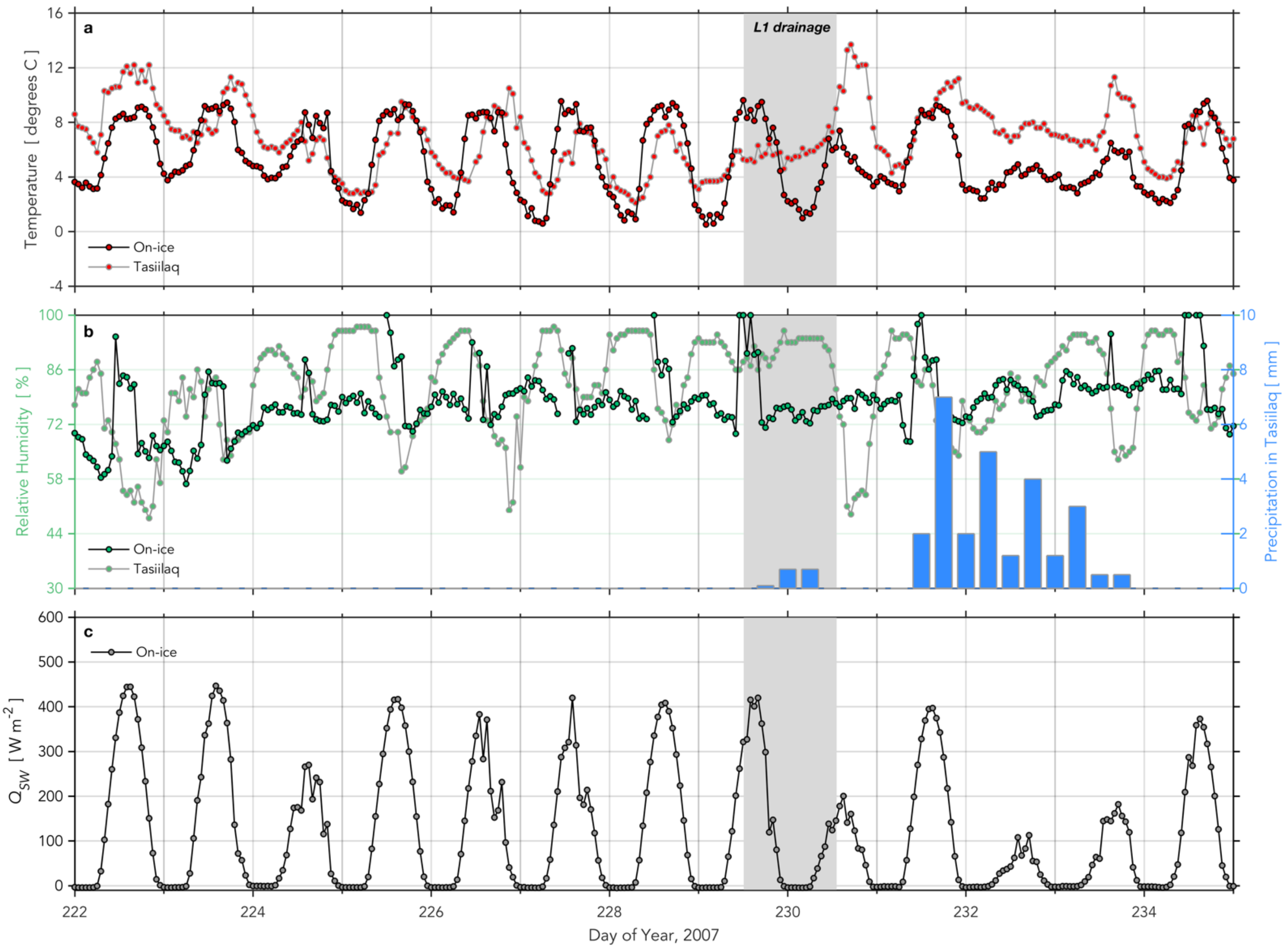

Supplementary Figure 5: Automatic Weather Station (AWS) observations show no anomalous temperature, insolation, or precipitation immediately prior to 2007 lake-drainage event. AWS observations from (bold hues) an on-ice AWS at Helheim Glacier (Fig. 1a; ref. 11) and (greyed hues) a land-based AWS in Tasiilaq[11] of (**a**) temperature and (**b**, left axis) relative humidity. Grey shading shows L1 drainage event from t_0 at station IS27 to t_{node} averaged across stations IS35–39. (**b**, right axis) Precipitation in Tasiilaq. (**c**) On-ice AWS observations of net short-wave radiative flux Q_{SW} (insolation), the AWS variable most closely correlated with the total energy flux available for melting[12]. Source data are provided as a source data file.

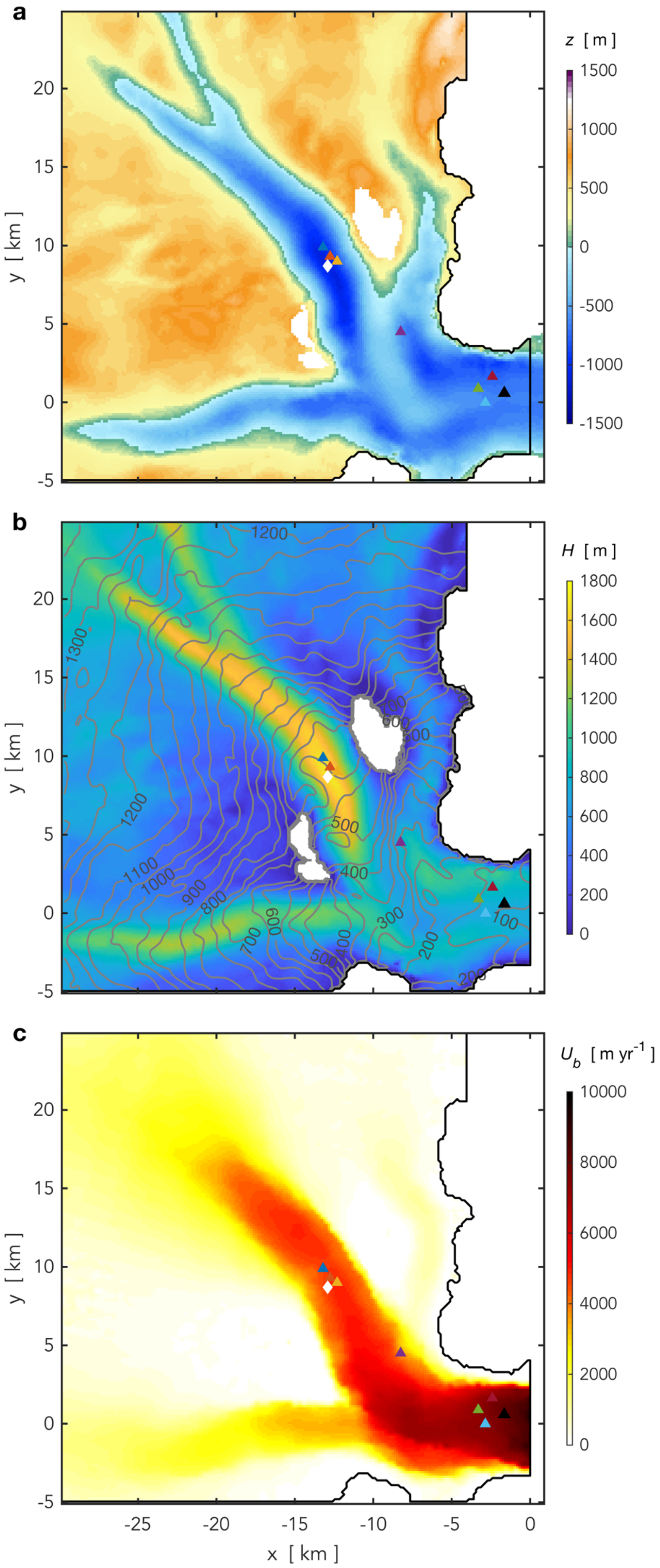

Supplementary Figure 6: Model domain. (**a**) Bedrock topography z relative to the WGS84 ellipsoid, (**b**) ice-sheet thickness H, and (**c**) basal sliding speed U_b of the model domain. Black outline is the edge of the model domain. White diamond shows location of simulated lake drainage location. Triangles show the locations of GPS stations. Pixelated look of values in all panels shows the 150-m-resolution model grid. In panel **b**, grey contour lines show ice-sheet surface elevation contours at 50-m intervals. Bedrock topography and glacier thickness in panels **a** and **b** are from BedMachine v3[13]. Basal sliding velocities in panel **c** are from the MEaSUREs Greenland Ice Sheet Velocity compilation[14,15] for July 2007. Source data are provided as a source data file.

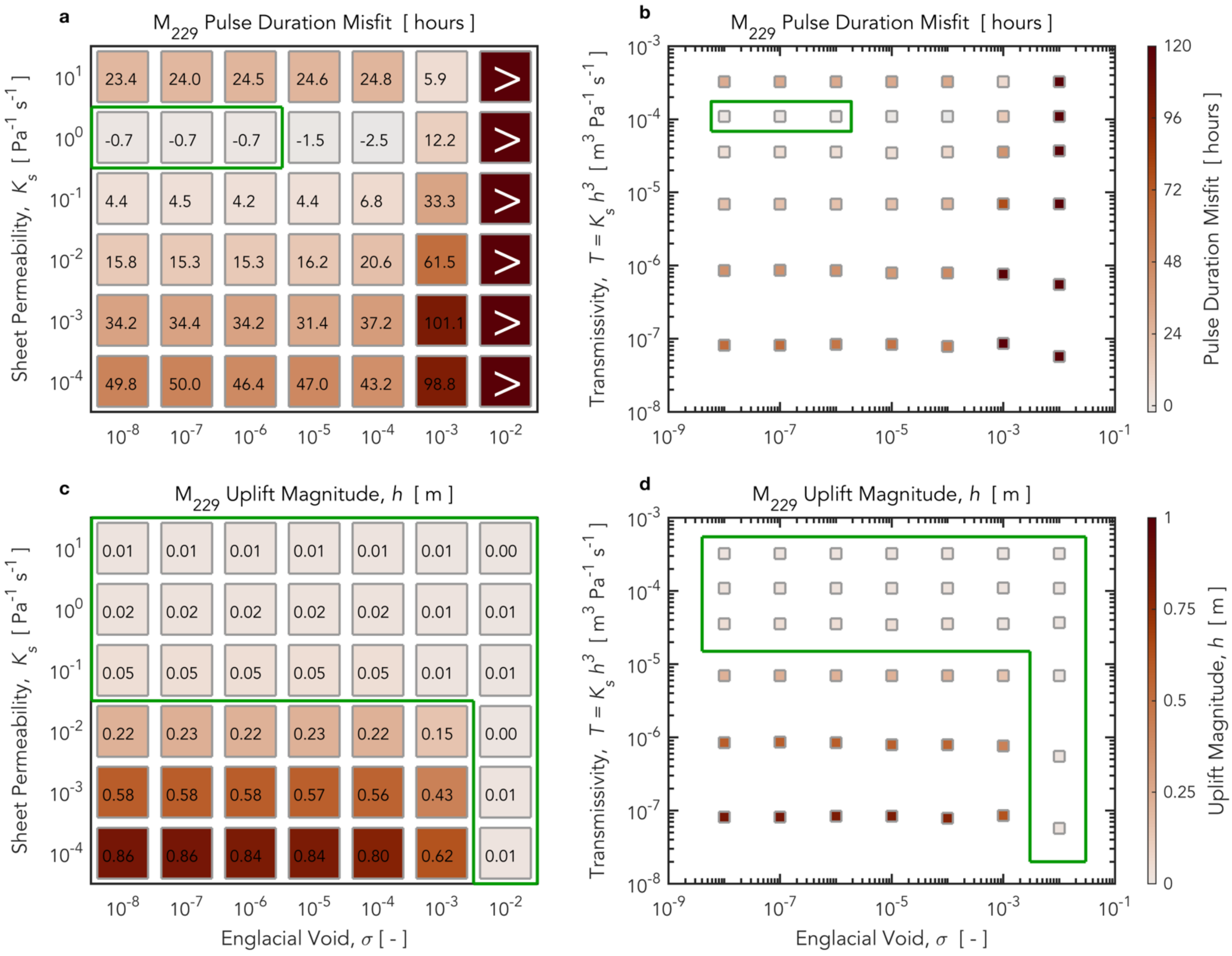

Supplementary Figure 7: Pulse-duration misfit and uplift magnitude for model parameter space. (**a**) (colors, black text) Pulse-duration misfit in hours between the observed velocity pulse duration and model results for M_{229} simulations spanning the parameter space for sheet permeability K_s and englacial void fraction σ. Green box encloses minimum-misfit simulations. White greater-than signs (>) are models where the duration of reduced effective pressures extends beyond the length of the model run, which ends 132 hours (5.5 days) after lake-drainage onset. **b**) Pulse-duration misfit plotted for the englacial-void σ parameter space and model transmissivity T, calculated as $K_s h^3$, where h is the height of the sheet[16] at the location of L1 one day prior to the lake drainage. Green box encloses minimum-misfit simulations. Color scale is equivalent in panels **a** and **b**. (**c, d**) Equivalent plots for uplift magnitude, where uplift magnitude in model simulations is calculated as the maximum height change of the sheet layer during the simulated lake drainage at the location of IS27–29 in the model domain. Green box encloses region of the parameter space where uplift magnitude does not exceed 0.09 m, equivalent to 3σ uncertainty in vertical positions associated with the TRACK position solutions. Color scale is equivalent in panels **c** and **d**. Models that satisfy both pulse duration and uplift magnitude criteria are found within a well-sampled region of parameter space in T, where $T \sim 10^{-4}$ m^3 Pa^{-1} s^{-1}. Source data are provided as a source data file.

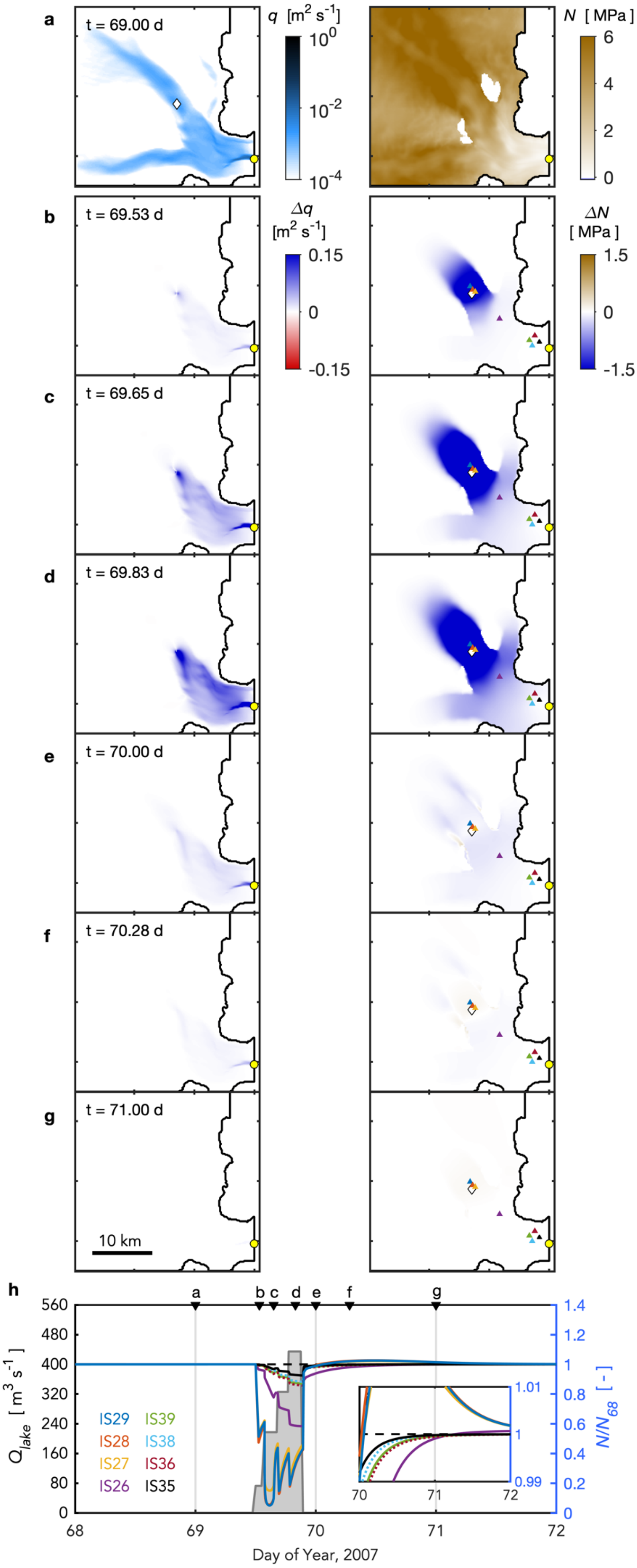

Supplementary Figure 8: Modeled effective-pressure response to M_{winter} simulated lake drainage. Panels are as in Fig. 3, but for an M_{winter} simulation. (**a**) (left) Discharge q and (right) effective pressure N prior to rapid lake drainage. Yellow circle shows discharge outlet location along the glacier terminus. White diamond shows location of simulated lake drainage. (**b–g)** Difference between modeled values of q and N at six time points during the simulated lake drainage and the model values shown in a at 2007/69.00 and prior to the start of the simulated lake drainage. Triangles show GPS station locations. (**h**) (grey shading) Prescribed lake discharge Q_{lake} and (curves) modeled effective pressure at the location of each GPS station, plotted as N/N_{68}, where N_{68} are individual average values of N at each GPS station location on 2007/68. Black triangles mark time slices shown in **a–g**. (**i**) Modeled effective pressure from 2007/230–232 at the location of each GPS station, plotted as N/N_{68}, over a finer range in N/N_{68} than shown in **h**. Model simulation uses parameter values K_s = 1 Pa^{-1} s^{-1} and σ = 10^{-6}. Source data are provided as a source data file.

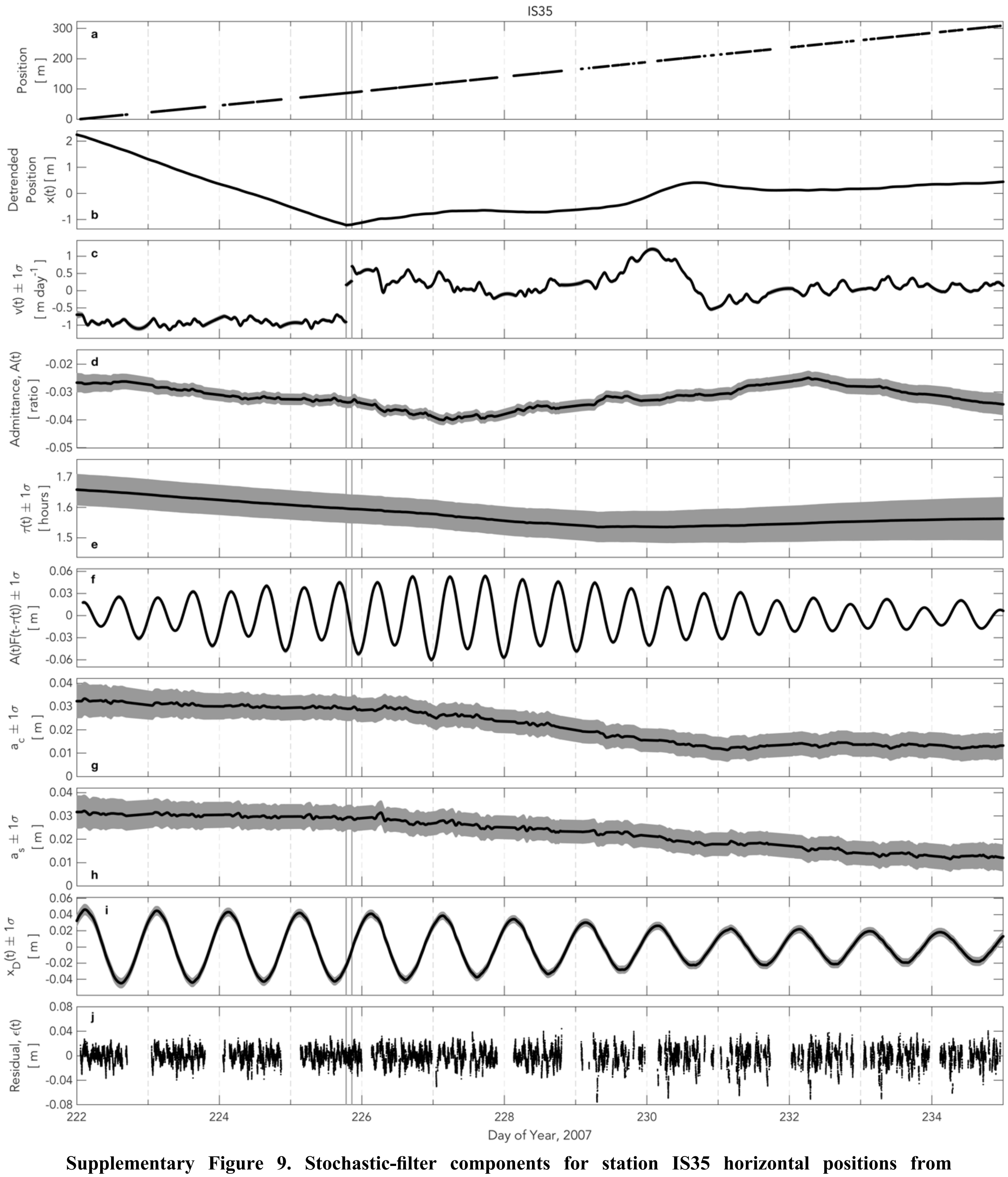

Supplementary Figure 9. Stochastic-filter components for station IS35 horizontal positions from 2007/222–235. (**a**) Along-flow station position; (**b**) detrended along-flow position $x(t)$; (**c**) non-periodic along-flow speed $v(t)$; (**d**) ocean tidal admittance $A(t)$; (**e**) lag in tidal response, $\tau(t)$; (**f**) estimated horizontal glacier response to ocean tide, $A(t)F\big(t-\tau(t)\big)$, from values shown in panels **d** and **e**; (**g**) stochastic amplitude $a_c(t)$; (**h**) stochastic amplitude $a_s(t)$; (**i**) estimated horizontal diurnal variation in glacier position, $x_D(t)$, from values shown in panels **g** and **h**; and, (**j**) model residual $\epsilon(t)$. Grey shading shows ±1σ error bounds. Vertical grey lines show times of glacial earthquakes. Data gaps resulting from elimination of noisy data are visible in the position (**a**) and residual (**j**); the modeled values are continuous.

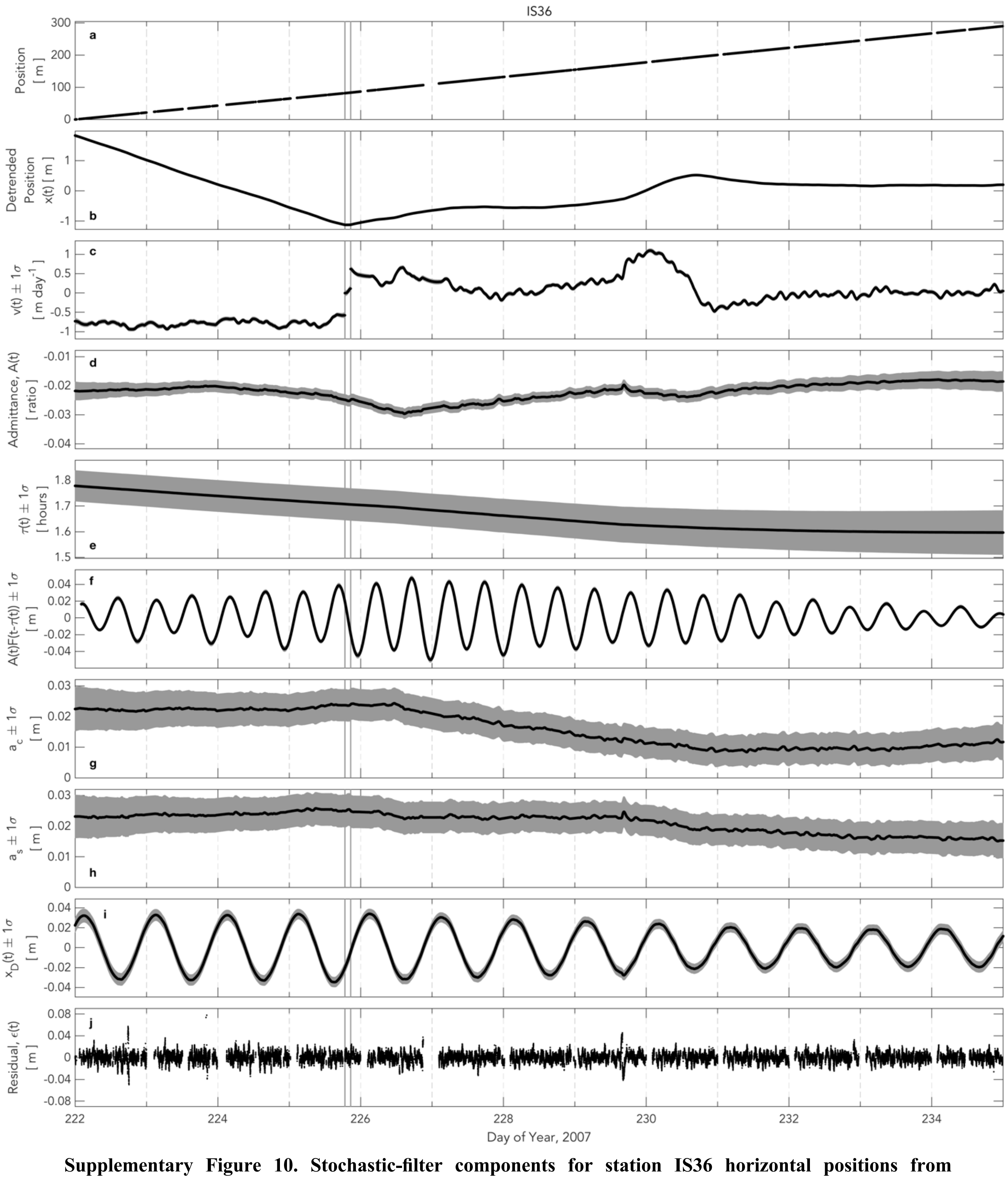

Supplementary Figure 10. Stochastic-filter components for station IS36 horizontal positions from 2007/222–235. (**a**) Along-flow station position; (**b**) detrended along-flow position $x(t)$; (**c**) non-periodic along-flow speed $v(t)$; (**d**) ocean tidal admittance $A(t)$; (**e**) lag in tidal response, $\tau(t)$; (**f**) estimated horizontal glacier response to ocean tide, $A(t)F\big(t-\tau(t)\big)$, from values shown in panels **d** and **e**; (**g**) stochastic amplitude $a_c(t)$; (**h**) stochastic amplitude $a_s(t)$; (**i**) estimated horizontal diurnal variation in glacier position, $x_D(t)$, from values shown in panels **g** and **h**; and, (**j**) model residual $\epsilon(t)$. Grey shading shows ±1σ error bounds. Vertical grey lines show times of glacial earthquakes. Data gaps resulting from elimination of noisy data are visible in the position (**a**) and residual (**j**); the modeled values are continuous.

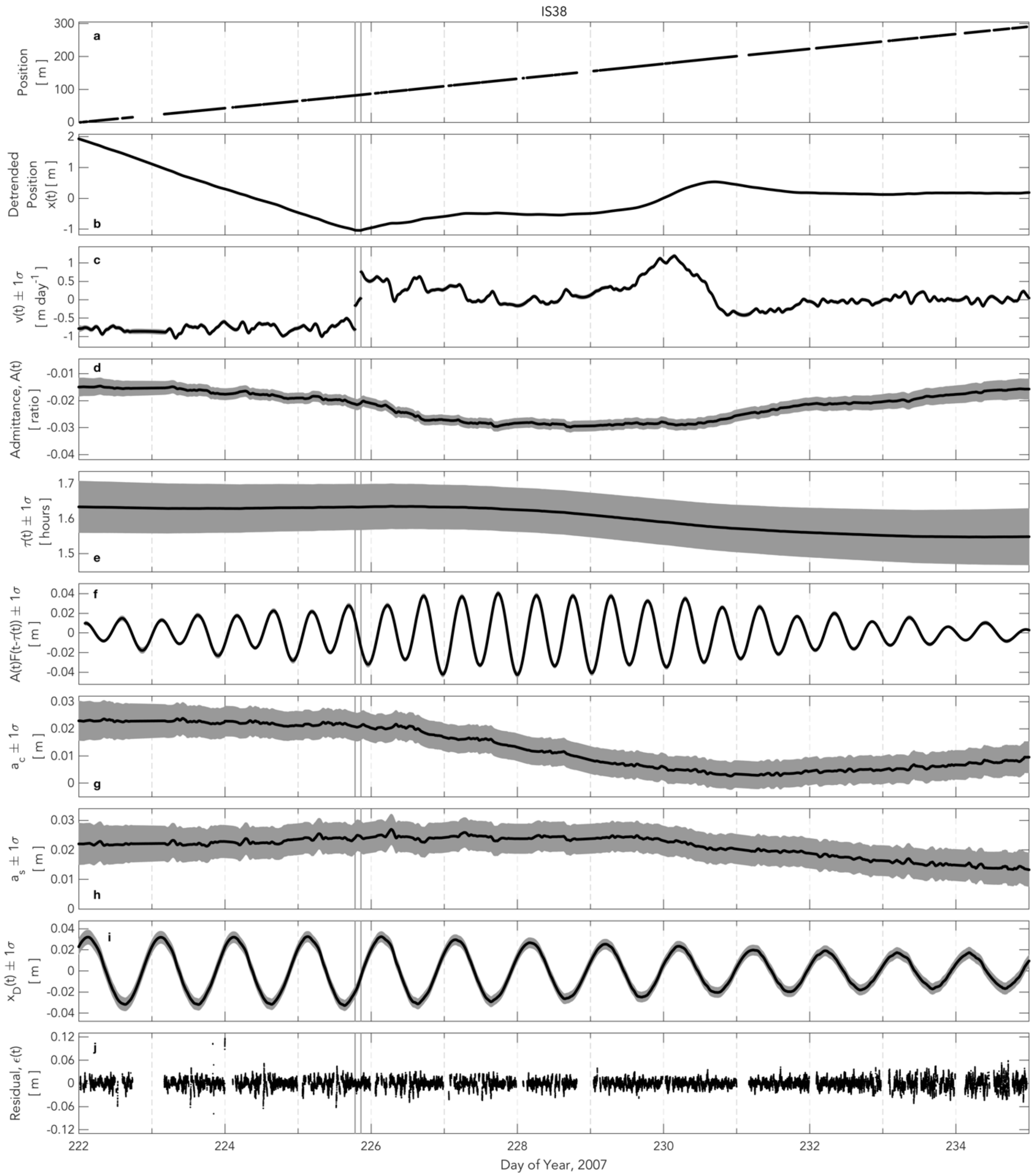

Supplementary Figure 11. Stochastic-filter components for station IS38 horizontal positions from 2007/222–235. (**a**) Along-flow station position; (**b**) detrended along-flow position $x(t)$; (**c**) non-periodic along-flow speed $v(t)$; (**d**) ocean tidal admittance $A(t)$; (**e**) lag in tidal response, $\tau(t)$; (**f**) estimated horizontal glacier response to ocean tide, $A(t)F\big(t-\tau(t)\big)$, from values shown in panels **d** and **e**; (**g**) stochastic amplitude $a_c(t)$; (**h**) stochastic amplitude $a_s(t)$; (**i**) estimated horizontal diurnal variation in glacier position, $x_D(t)$, from values shown in panels **g** and **h**; and, (**j**) model residual $\epsilon(t)$. Grey shading shows ±1σ error bounds. Vertical grey lines show times of glacial earthquakes. Data gaps resulting from elimination of noisy data are visible in the position (**a**) and residual (**j**); the modeled values are continuous.

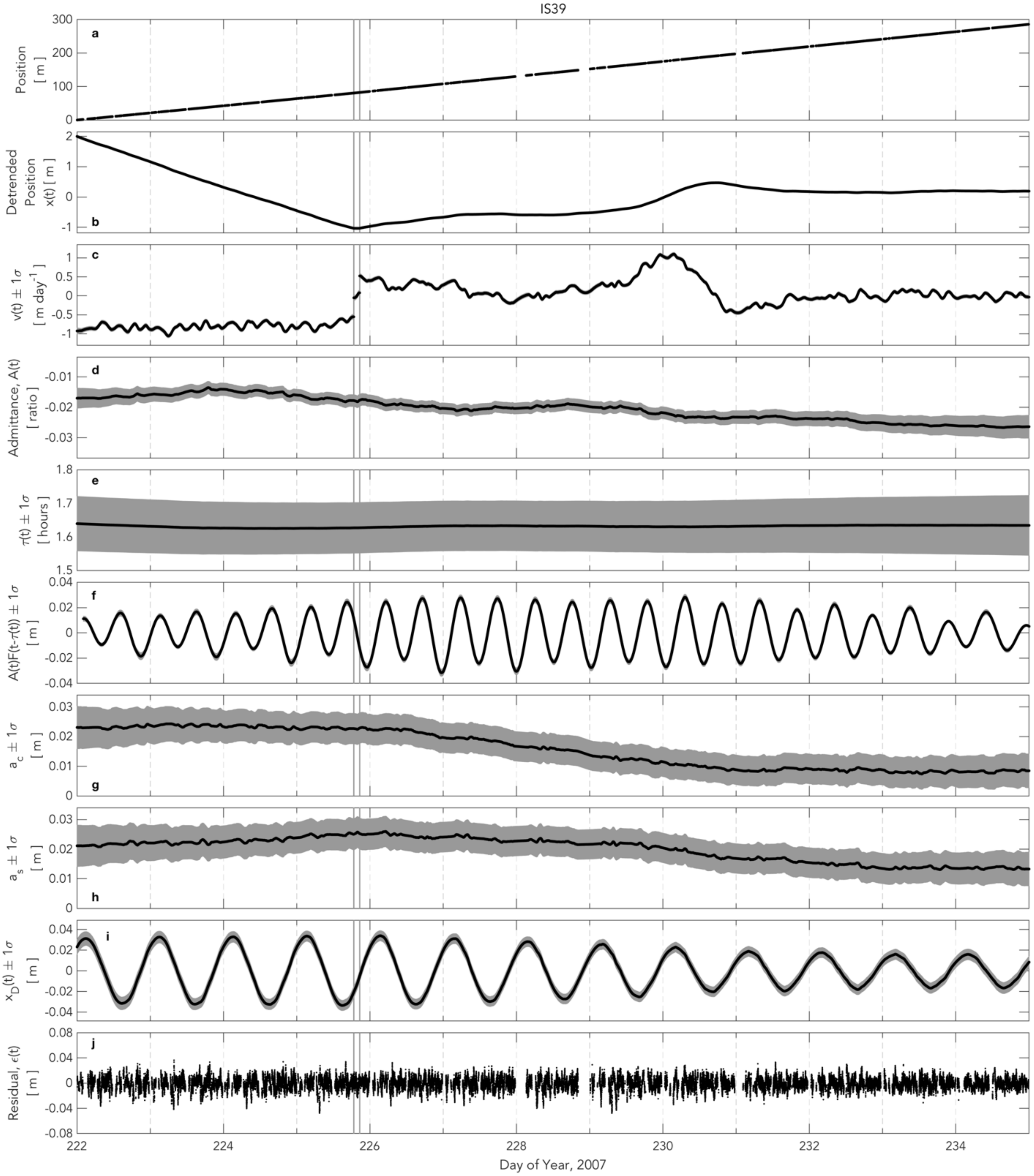

Supplementary Figure 12. Stochastic-filter components for station IS39 horizontal positions from 2007/222–235. (**a**) Along-flow station position; (**b**) detrended along-flow position $x(t)$; (**c**) non-periodic along-flow speed $v(t)$; (**d**) ocean tidal admittance $A(t)$; (**e**) lag in tidal response, $\tau(t)$; (**f**) estimated horizontal glacier response to ocean tide, $A(t)F\big(t-\tau(t)\big)$, from values shown in panels **d** and **e**; (**g**) stochastic amplitude $a_c(t)$; (**h**) stochastic amplitude $a_s(t)$; (**i**) estimated horizontal diurnal variation in glacier position, $x_D(t)$, from values shown in panels **g** and **h**; and, (**j**) model residual $\epsilon(t)$. Grey shading shows ±1σ error bounds. Vertical grey lines show times of glacial earthquakes. Data gaps resulting from elimination of noisy data are visible in the position (**a**) and residual (**j**); the modeled values are continuous.

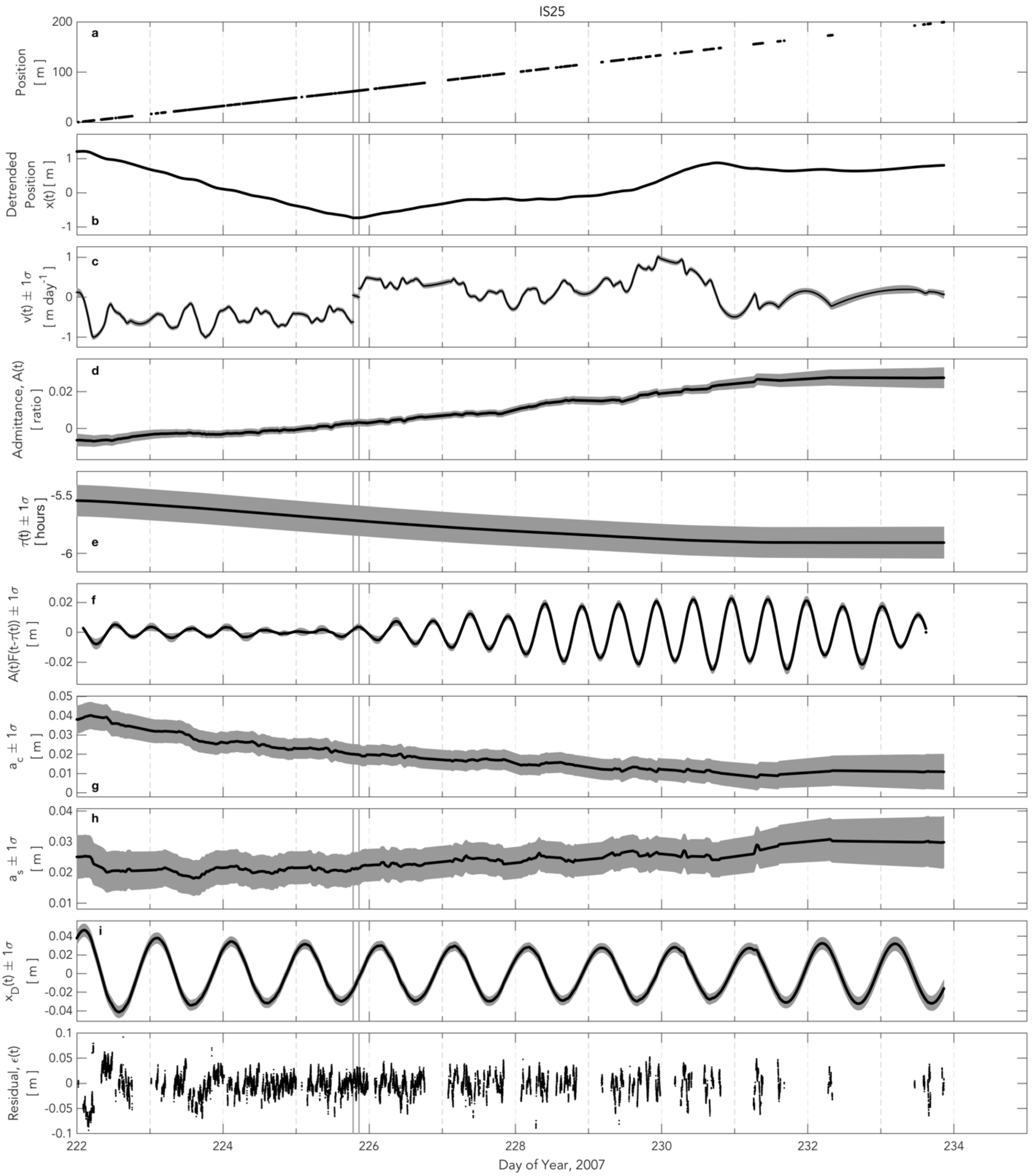

Supplementary Figure 13. Stochastic-filter components for station IS25 horizontal positions from 2007/222–235. (**a**) Along-flow station position; (**b**) detrended along-flow position $x(t)$; (**c**) non-periodic along-flow speed $v(t)$; (**d**) ocean tidal admittance $A(t)$; (**e**) lag in tidal response, $\tau(t)$; (**f**) estimated horizontal glacier response to ocean tide, $A(t)F\big(t-\tau(t)\big)$, from values shown in panels **d** and **e**; (**g**) stochastic amplitude $a_c(t)$; (**h**) stochastic amplitude $a_s(t)$; (**i**) estimated horizontal diurnal variation in glacier position, $x_D(t)$, from values shown in panels **g** and **h**; and, (**j**) model residual $\epsilon(t)$. Grey shading shows ±1σ error bounds. Vertical grey lines show times of glacial earthquakes. Data gaps resulting from elimination of noisy data are visible in the position (**a**) and residual (**j**); the modeled values are continuous.

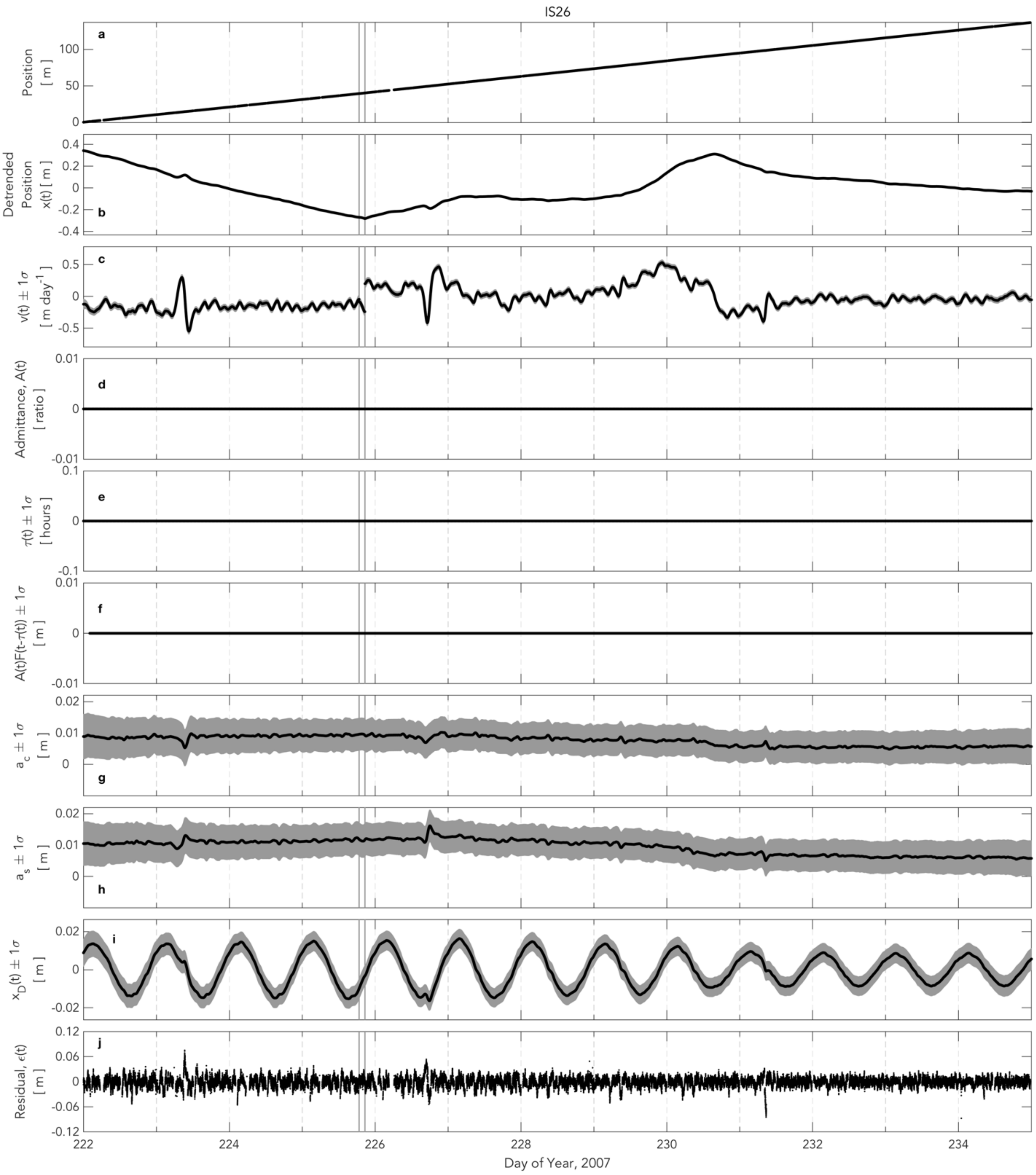

Supplementary Figure 14. Stochastic-filter components for station IS26 horizontal positions from 2007/222–235. (**a**) Along-flow station position; (**b**) detrended along-flow position $x(t)$; (**c**) non-periodic along-flow speed $v(t)$; (**d**) ocean tidal admittance $A(t)$; (**e**) lag in tidal response, $\tau(t)$; (**f**) estimated horizontal glacier response to ocean tide, $A(t)F\big(t-\tau(t)\big)$, from values shown in panels **d** and **e**; (**g**) stochastic amplitude $a_c(t)$; (**h**) stochastic amplitude $a_s(t)$; (**i**) estimated horizontal diurnal variation in glacier position, $x_D(t)$, from values shown in panels **g** and **h**; and, (**j**) model residual $\epsilon(t)$. Grey shading shows $\pm 1\sigma$ error bounds. Vertical grey lines show times of glacial earthquakes. Data gaps resulting from elimination of noisy data are visible in the position (**a**) and residual (**j**); the modeled values are continuous.

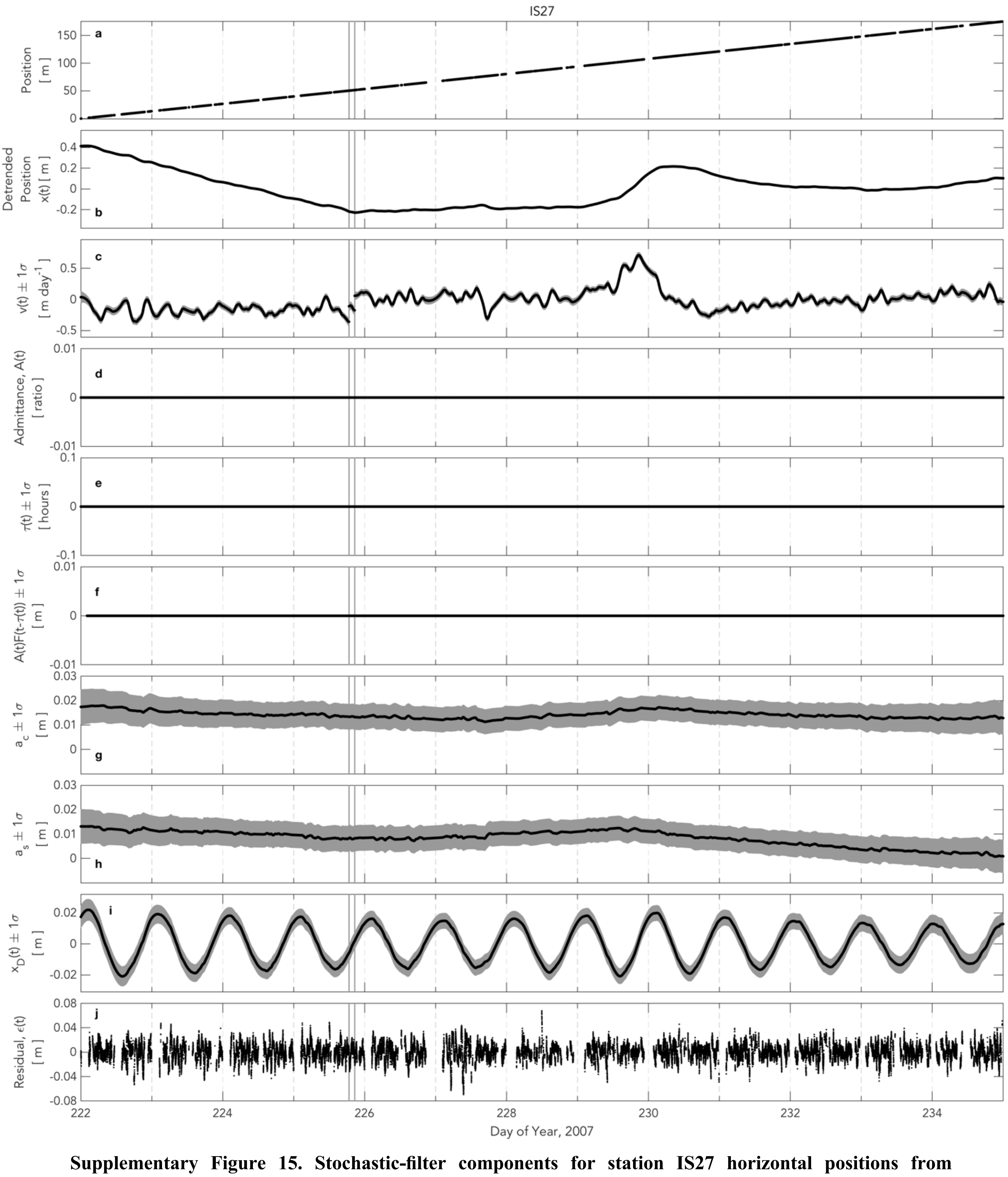

Supplementary Figure 15. Stochastic-filter components for station IS27 horizontal positions from 2007/222–235. (**a**) Along-flow station position; (**b**) detrended along-flow position $x(t)$; (**c**) non-periodic along-flow speed $v(t)$; (**d**) ocean tidal admittance $A(t)$; (**e**) lag in tidal response, $\tau(t)$; (**f**) estimated horizontal glacier response to ocean tide, $A(t)F\big(t-\tau(t)\big)$, from values shown in panels **d** and **e**; (**g**) stochastic amplitude $a_c(t)$; (**h**) stochastic amplitude $a_s(t)$; (**i**) estimated horizontal diurnal variation in glacier position, $x_D(t)$, from values shown in panels **g** and **h**; and, (**j**) model residual $\epsilon(t)$. Grey shading shows ±1σ error bounds. Vertical grey lines show times of glacial earthquakes. Data gaps resulting from elimination of noisy data are visible in the position (**a**) and residual (**j**); the modeled values are continuous.

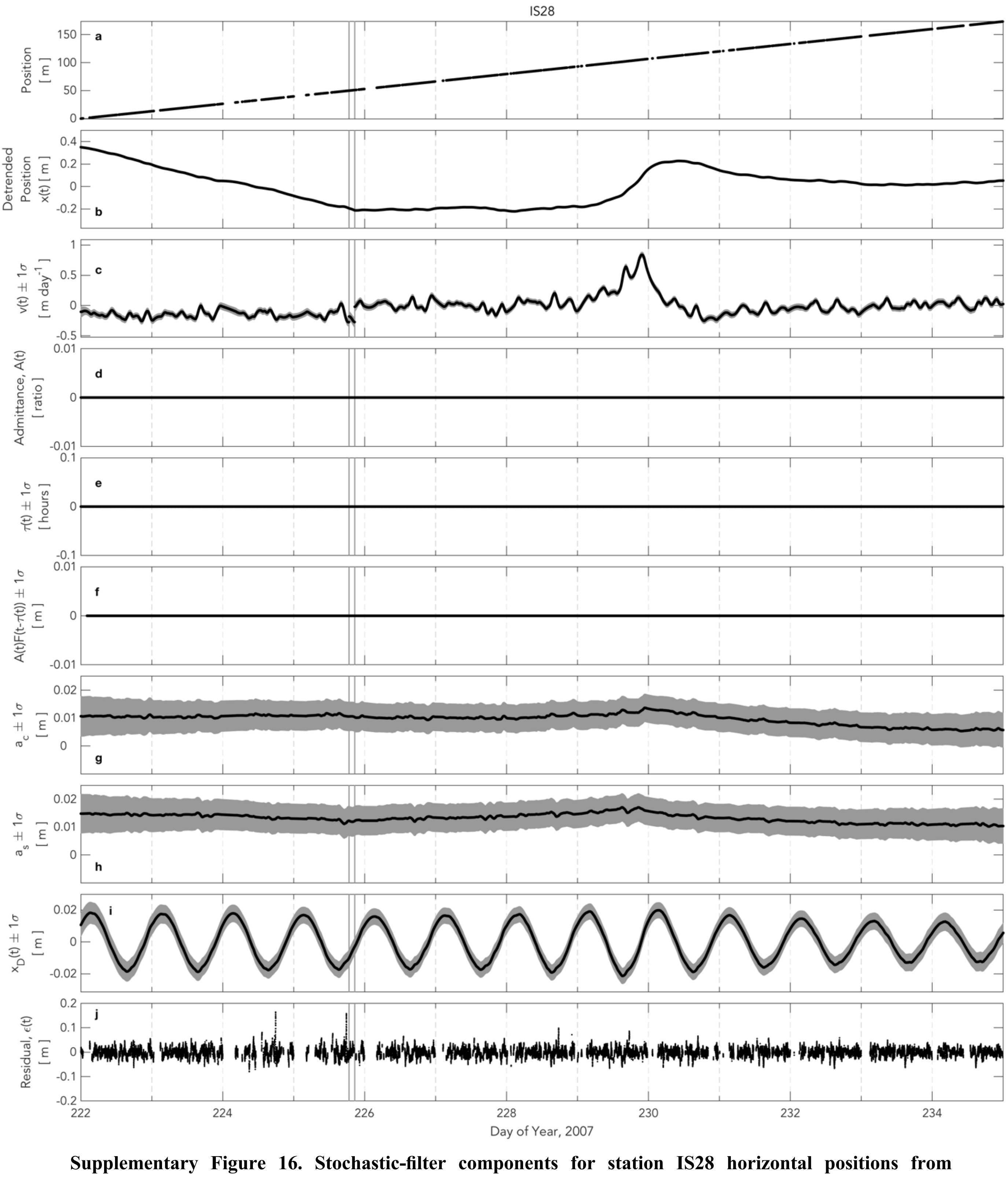

Supplementary Figure 16. Stochastic-filter components for station IS28 horizontal positions from 2007/222–235. (**a**) Along-flow station position; (**b**) detrended along-flow position $x(t)$; (**c**) non-periodic along-flow speed $v(t)$; (**d**) ocean tidal admittance $A(t)$; (**e**) lag in tidal response, $\tau(t)$; (**f**) estimated horizontal glacier response to ocean tide, $A(t)F\big(t-\tau(t)\big)$, from values shown in panels **d** and **e**; (**g**) stochastic amplitude $a_c(t)$; (**h**) stochastic amplitude $a_s(t)$; (**i**) estimated horizontal diurnal variation in glacier position, $x_D(t)$, from values shown in panels **g** and **h**; and, (**j**) model residual $\epsilon(t)$. Grey shading shows ±1σ error bounds. Vertical grey lines show times of glacial earthquakes. Data gaps resulting from elimination of noisy data are visible in the position (**a**) and residual (**j**); the modeled values are continuous.

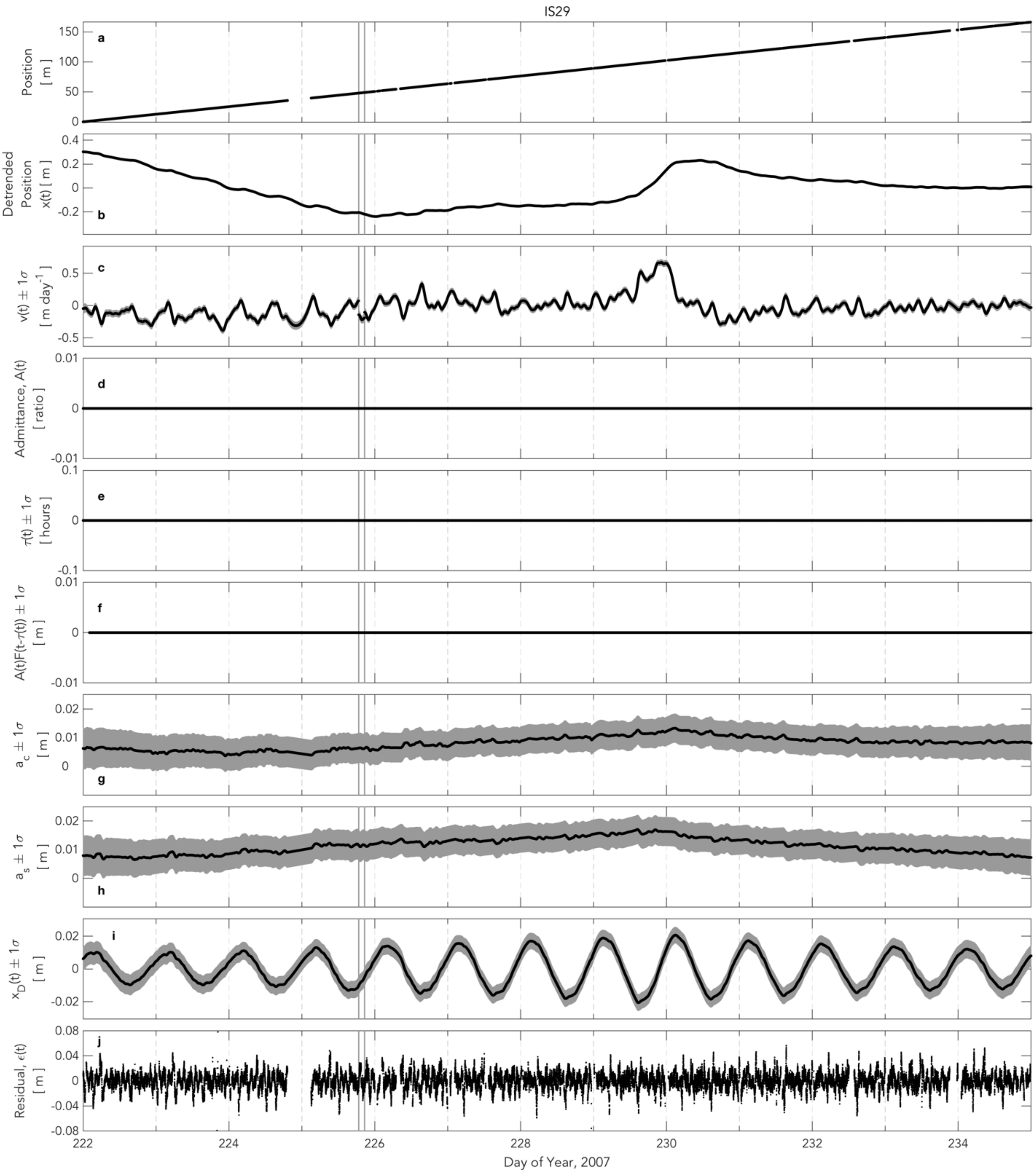

Supplementary Figure 17. Stochastic-filter components for station IS29 horizontal positions from 2007/222–235. (**a**) Along-flow station position; (**b**) detrended along-flow position $x(t)$; (**c**) non-periodic along-flow speed $v(t)$; (**d**) ocean tidal admittance $A(t)$; (**e**) lag in tidal response, $\tau(t)$; (**f**) estimated horizontal glacier response to ocean tide, $A(t)F\big(t-\tau(t)\big)$, from values shown in panels **d** and **e**; (**g**) stochastic amplitude $a_c(t)$; (**h**) stochastic amplitude $a_s(t)$; (**i**) estimated horizontal diurnal variation in glacier position, $x_D(t)$, from values shown in panels **g** and **h**; and, (**j**) model residual $\epsilon(t)$. Grey shading shows ±1σ error bounds. Vertical grey lines show times of glacial earthquakes. Data gaps resulting from elimination of noisy data are visible in the position (**a**) and residual (**j**); the modeled values are continuous.

3. Supplementary Table

Supplementary Table 1: Subglacial-hydrology model: parameter values and ranges.

Variable	Description	Value
ρ_w	Water density	1000 kg m^{-3}
ρ_i	Ice density	910 kg m^{-3}
g	Gravitational acceleration	9.8 m s^{-2}
A	Glen's law fluidity coefficient	6.8×10^{-24} Pa^{-3} s^{-1}
n	Glen's law exponent	3
L	Latent heat of melting	3.5×10^{5} J kg^{-3}
G	Greenland geothermal heat flux	0.063 W m^{-2} *
m	Basal melt rate	0.0262 m yr^{-1}
σ	Englacial void fraction	[10^{-8},10^{-7},10^{-6},10^{-5},10^{-4},10^{-3},10^{-2}]
K_c	Turbulent flow coefficient for channel flow	0.1 m s^{-1} Pa$^{-1/2}$
K_s	Sheet flux coefficient (sheet permeability)	[10^{-4},10^{-3},10^{-2},10^{-1},10^{0},10^{1}] Pa^{-1} s^{-1}
λ_c	Sheet width contributing to melting	1000 m
c	Specific heat capacity of water	4200 J kg^{-1} K^{-1}
β	Melting point pressure gradient	7.8×10^{-8} K Pa^{-1}
h_r	Bed roughness height scale	0.1 m
l_r	Bed roughness length scale	10 m
U_b	Basal sliding speed	0–10^{4} m yr^{-1} **
C_{el}	Uplift regularization rate	1.02×10^{-6} m Pa^{-1}

* value from ref. 17
** values from MEaSUREs Greenland Ice Sheet Velocity compilation[14,15] for July 2007

References

1. Chen, G. GPS kinematic positioning for the airborne laser altimetry at Long Valley, California. (Massachusetts Institute of Technology, Cambridge, Massachusetts, 1998).
2. Nettles, M. *et al.* Step-wise changes in glacier flow speed coincide with calving and glacial earthquakes at Helheim Glacier, Greenland. *Geophys. Res. Lett.* **35**, L24503 (2008).
3. de Juan, J. *et al.* Sudden increase in tidal response linked to calving and acceleration at a large Greenland outlet glacier. *Geophys. Res. Lett.* **37**, L12501 (2010).
4. Davis, J. L., de Juan, J., Nettles, M., Elósegui, P. & Andersen, M. L. Evidence for non-tidal diurnal velocity variations of Helheim Glacier, East Greenland. *J. Glaciol.* **60**, 1221–1231 (2014).
5. Stevens, L. A. *et al.* Helheim Glacier diurnal velocity fluctuations driven by surface melt forcing. *J. Glaciol.* **68**, 77–89 (2021).
6. Ravishanker, N. & Dey, D. K. *A first course in linear model theory*. (CRC Press, 2002).
7. Davis, J. L., Wernicke, B. P. & Tamisiea, M. E. On seasonal signals in geodetic time series. *J. Geophys. Res. Solid Earth* **117**, 1–10 (2012).
8. Padman, L. & Erofeeva, S. A barotropic inverse tidal model for the Arctic Ocean. *Geophys. Res. Lett.* **31**, L02303 (2004).
9. Sohn, D. H., Park, K. D., Davis, J. L., Nettles, M. & Elósegui, P. Rapid ionospheric variations at high latitudes: Focusing on Greenland. *Adv. Sp. Res.* **65**, 1673–1684 (2020).
10. Everett, A. *et al.* Annual down-glacier drainage of lakes and water-filled crevasses at Helheim Glacier, southeast Greenland. *J. Geophys. Res. Earth Surf.* **121**, 1819–1833 (2016).
11. Cappelen, J. *Technical Report 14-08 Weather observations from Greenland 1958-2013: Observation data with description*. (2014).
12. Andersen, M. L. *et al.* Spatial and temporal melt variability at Helheim Glacier, East Greenland, and its effect on ice dynamics. *J. Geophys. Res. Earth Surf.* **115**, 1–18 (2010).
13. Morlighem, M., Rignot, E., Mouginot, J., Seroussi, H. & Larour, E. Deeply incised submarine glacial valleys beneath the Greenland ice sheet. *Nat. Geosci.* **7**, 18–22 (2014).
14. Joughin, I., Smith, B. & Howat, I. A complete map of Greenland ice velocity derived from satellite data collected over 20 years. *J. Glaciol.* 1–11 (2018).
15. Joughin, I., Smith, B. E. & Howat, I. Greenland Ice Mapping Project: Ice flow velocity variation at sub-monthly to decadal time scales. *Cryosph.* **12**, 2211–2227 (2018).
16. Hewitt, I. J. Seasonal changes in ice sheet motion due to melt water lubrication. *Earth Planet. Sci. Lett.* **371**, 16–25 (2013).
17. Rogozhina, I. *et al.* Effects of uncertainties in the geothermal heat flux distribution on the Greenland Ice Sheet: An assessment of existing heat flow models. *J. Geophys. Res.* **117**, F02025 (2012).
